# Person-Centered Study of Cognitive Ability Dimensions Using Latent Profile Analysis

**DOI:** 10.3390/jintelligence11050080

**Published:** 2023-04-26

**Authors:** Jeffrey M. Conte, Rebecca K. Harmata

**Affiliations:** 1 Department of Psychology, San Diego State University, San Diego, CA 92182, USA; 2 Department of Psychology, University of Georgia, Athens, GA 30602, USA

**Keywords:** person-centered approach, cognitive ability dimensions, latent profile analysis

## Abstract

A number of researchers have called for additional investigations into cognitive ability and intelligence in recent years. This paper utilized a person-centered approach, multiple cognitive ability dimensions, and latent profile analysis to investigate multivariate relationships among cognitive ability dimensions in a sample of 1681 Army recruits. Six cognitive ability dimensions were assessed via the Armed Services Vocational Aptitude Battery. Performance measures were obtained from supervisor ratings of Effort, Discipline, and Peer Leadership. Using latent profile analysis, the results identified five distinct cognitive profiles or classes, which differed significantly across the three types of supervisor ratings.

## 1. Introduction

A number of researchers have called for additional investigations of cognitive ability and intelligence in recent years. In a focal article in *Industrial and Organizational Psychology: Perspectives on Science and Practice*, Scherbaum et al. (2012) noted that although the field of industrial and organizational (I–O) psychology has not studied the intelligence construct in depth in a number of years, other fields (e.g., clinical, educational, and developmental) have continued to study this construct and have made substantial progress in understanding it in more detail. Scherbaum and colleagues challenged the field of I–O psychology to pursue new research initiatives on this critical construct. 

As noted by Conte and Landy (2019), people use cognitive abilities to acquire knowledge and solve problems. Studies across many decades of research indicate that cognitive ability is one of the best predictors of job performance (e.g., Sackett et al. 2022; Schmidt and Hunter 1998). Further, meta-analyses investigating the relationship between general intelligence and job performance have demonstrated that as the complexity and cognitive demands of jobs increase, the predictive validity of tests of general intelligence also increases (Schmidt and Hunter 2004).

Schneider and Newman (2015) reviewed a wide array of research to support their claim that intelligence is a multidimensional construct. They recommended a renewed interest in narrower cognitive abilities, as compared to the unidimensional view captured by “general mental ability”—or, simply, “g”. Reeve et al. (2015) similarly noted that although the focus on “g” continues to provide insight into successful behavior at work, there is a need to give additional consideration of specific aspects of intelligence. They argued that giving attention to *constellations* of specific cognitive aptitudes can provide additional insight into the various abilities that are required for success in today’s workplace. Thus, their article encouraged I–O psychologists to use the science of mental abilities and measurement theory to better understand how basic constructs within the intelligence literature affect job performance. 

In the developmental psychology area, Fuchs et al. (2010) examined whether different types of school mathematics development depend on different constellations of numerical versus general cognitive abilities. First graders (n = 280) were assessed on two types of basic numerical cognition, eight domain-general abilities, procedural calculations (PCs), and word problems (WPs) in the Fall, and then reassessed on PCs and WPs in the Spring. The results suggested that the development of different types of formal school mathematics depends on various constellations of numerical versus general cognitive abilities. 

This study used multiple cognitive ability dimensions and the person-centered approach to identify cognitive profiles and to predict supervisory ratings of job performance. It used a multidisciplinary approach by considering research and theories on cognitive ability from educational, developmental, cognitive, and I–O psychology to investigate multivariate relationships between cognitive ability dimensions and performance outcomes. 

### 1.1. Concerns about Positive Manifold of Cognitive Ability

Any study of multiple cognitive ability dimensions naturally should address concerns about the fact that positive manifold (correlations) found in specific measures of cognitive ability might limit the utility of class-based approaches, such as Latent Profile Analysis (LPA). There are several reasons why exploring multiple dimensions of cognitive ability using class-based approaches such as LPA can be beneficial and informative. First, dominant general factors and positive manifold have been found in variety of constructs beyond cognitive ability, including in constructs (personality, emotional intelligence) that have successfully used class-based analyses. 

Ree et al. (2015) noted that positive manifold is common among many constructs (not just cognitive ability). For example, they present evidence that the following constructs show positive manifold and a dominant general factor (DGF): emotional intelligence, personality, psychomotor ability, job performance, job satisfaction, core self-evaluation, leadership, and physical ability. Ree and colleagues indicated that the percentage of variance accounted for by the DGF for cognitive ability measures ranged from 41% to 64%, whereas the percentage of variance accounted for by the DGF for personality measures ranged from 40% to 50%. Another construct that has multiple dimensions and has been investigated with class-based approaches is emotional intelligence (Keefer et al. 2012). Ree and colleagues noted that the percentage of variance accounted for by DGF for EI measures ranges from 52% to 70%. So, although for example, personality and emotional intelligence have dominant general factors and positive manifold, researchers have nevertheless been able to produce meaningfully different sets of classes or profiles with different dimensions of personality and emotional intelligence. 

Studies examining specific cognitive ability dimensions should be conducted despite positive manifold, which has previously led to a lack of research in I–O psychology on specific dimensions of cognitive ability, despite the important progress that other areas of psychology (such as clinical, developmental, and educational psychology) have made in this area.

### 1.2. Person-Centered Approach

An issue of the journal *Industrial and Organizational Psychology: Perspectives on Science and Practice* included a focal article and several commentaries on person-centric psychology (Weiss and Rupp 2011). In contrast to the prevailing paradigm in I–O psychology that focuses on variables and relationships between variables, person-centric psychology focuses on the person, and takes a holistic and dynamic view of people (Foti et al. 2011). Person-centric approaches utilize multiple individual difference characteristics or dimensions to identify profiles or patterns of individuals who are similar (Wang et al. 2013). 

The person-centered approach has been used with personality variables (Asendorpf 2015), but very little (if any) research has adopted this approach in the cognitive ability or intelligence domain. This study uses a person-centered approach to examine multiple dimensions of cognitive ability and how they relate to several performance outcomes. This study uses Latent Profile Analysis (LPA), which is a relatively new statistical approach that identifies homogeneous groups based on a number of predictor variables, such as cognitive ability dimensions. In particular, LPA (described further below) can be used to determine whether interactions between different cognitive dimensions can help organize respondents into groups with similar profiles. The identification of profile groups using Latent Profile Analysis can reveal new multivariate relationships among predictors and criteria that can contribute to improving the selection and job classification of employees in organizations. 

### 1.3. Latent Profile Analysis

Latent profile analysis (LPA) is a multivariate approach that defines classes of people based on common characteristics (Merz and Roesch 2011; Spurk et al. 2020). It is an empirically driven method that allows researchers to examine multiple observed dimensions simultaneously to define these classes via maximum likelihood estimation. For example, LPA can use all observations from cognitive ability scales to define classes via a maximum likelihood estimation. The primary goal is to maximize the homogeneity within groups (i.e., individuals within a class/profile should look similar) and maximize the heterogeneity between groups (i.e., individuals in different classes/profile groups should look different). Models are estimated with classes added iteratively to determine which model provides the best fit to the data. Latent profile analysis also has the advantage of allowing the testing of variables that are antecedents or outcomes of the different classes and profile groups.

Many cognitive ability dimensions have been found to covary (Carroll 1993). When considering multiple cognitive ability dimensions as predictors, the number of higher-order interactions is quite high, which can result in statistical problems such as reduced statistical power (Cohen et al. 2003). Because it is often impractical to model all higher-order interactions of interest, person-centered statistical approaches such as LPA can be used to mimic higher-order interaction terms (Merz and Roesch 2011). Further, LPA can organize interactive effects as subtypes in a way that offers a brief and simple summary of complicated relationships (Herzberg and Roth 2006). For this study, LPA was used to derive categorical latent variables that represent classes of individuals who share similar cognitive ability profiles (Lanza et al. 2003).

Psychologists have spent a lot of time classifying attributes of individuals using, for example, the Big Five personality dimensions, but much less time in classifying people themselves (Robins and Tracy 2003). Latent profile analysis provides a way to classify people and to focus more holistically on types of people, and thus works well with the person-centered approach. An increasing number of studies have used personality dimensions to classify people, but few (if any) studies have used cognitive ability dimensions. Thus, this study is among the first to do so.

Although evaluating the first-order effects of the cognitive dimensions might be considered a reasonable way to examine how cognitive ability is related to work outcomes, this method overlooks empirical evidence that cognitive dimensions do not exist in isolation. That is, although simultaneously entering the multiple dimensions as predictors in a statistical model controls for common variance, these types of models are only informative with regard to the additive effects (i.e., first order effects) of these dimensions (Merz and Roesch 2011) and preclude the possibility that the multiple dimensions can be modeled as multiplicative effects (i.e., interactions). As noted earlier, when considering multiple dimensions as predictors, the number of higher-order interactions is quite high, which can result in reduced statistical power (Cohen et al. 2003). Because it is often impractical to model all higher-order interactions, person-centered statistical approaches such as LPA can be used to mimic higher-order interaction terms (Lanza et al. 2010; Spurk et al. 2020). 

## 2. Present Investigation

Research using LPA provides an innovative examination of cognitive ability profiles and their potential links to performance outcomes. In particular, examining cognitive profiles can provide an understanding of different configurations of cognitive abilities. Although general intelligence is important for the prediction of job performance, research indicates that specific cognitive abilities can play an important role in predicting job and career success (Park et al. 2007; Webb et al. 2007). Specifically, research has shown the value of specific abilities in predicting performance using primarily correlation and regression methods (Lang and Kell 2020; Lang et al. 2010; Nye et al. 2022; Wee et al. 2014). These regression methods use a variable-centered approach that is common in I–O psychology and the psychology field in general. This paper has extended this work by investigating multiple cognitive ability measures simultaneously with the adoption of a person-centered approach (Foti et al. 2011). A number of studies have used the person-centered approach (e.g., Gabriel et al. 2015; Grunschel et al. 2013; Meyer et al. 2013); however, none to our knowledge has used the person-centered approach in the cognitive ability domain.

Empirical research indicates that specific cognitive ability dimensions are relatively independent from each other, and thus, they are likely to contribute to differing cognitive profiles that have meaningfully different cognitive strengths (Carroll 1993; Fleishman and Reilly 1992). In particular, research indicates that the cognitive ability measure (Armed Services Vocational Aptitude Battery, or ASVAB) used in this study has relatively independent dimensions (Segall 2004; Welsh et al. 1990). 

Once profile groups based on cognitive ability dimensions are established, they can be examined in relation to the performance outcomes to assess for meaningful prediction based on these latent classes. Investigating such profiles using LPA is relatively new to I–O psychology, and there have been increased calls for such research (e.g., Foti et al. 2011). By adopting a person-centered view and utilizing a relatively new multivariate approach to investigate relationships between multiple cognitive dimensions, the LPA results can help enhance the understanding of ways individuals differ in cognitive ability and enhance performance prediction as well. In particular, LPA can help to identify and understand previously unobserved subpopulations (Wang and Hanges 2011), which may then help with making more efficient and accurate selection and classification decisions. Based on reviews of the literature, the following research questions will be examined:

RQ1: Does Latent Profile Analysis provide evidence of multiple classes/profiles based on cognitive ability dimensions?

RQ2: Do performance outcomes differ across the cognitive ability profiles?

## 3. Materials and Method

### 3.1. Sample and Measures

The sample for this study comes from data from the Army Research Institute’s Personnel Assessment Research Unit (Knapp and Heffner 2010). The sample comprises 1681 Army recruits. In terms of the gender breakdown, 151 (9%) were female, and 1530 (91%) were male. The LPA analyses included 6 of the 10 subdimensions from the Armed Services Vocational Aptitude Battery (ASVAB). This subset focuses on the non-overlapping dimensions that still generally cover the overall domain of cognitive ability. The ASVAB is split into 5 Knowledge subtests and 5 Ability subtests. The current study used 2 dimensions from Knowledge subtests (General Science, Mechanical Comprehension) and 4 dimensions from the Ability subtests (Verbal Expression, Arithmetic Reasoning, Paragraph Comprehension, and Assembling Objects). From the original 10 ASVAB dimensions, this approach resulted in dropping Word Knowledge (which was correlated 0.93 with Verbal Expression) and Math Knowledge (which was correlated 0.51 with Arithmetic Reasoning). Other dimensions that were dropped were Automotive Shop and Electronics Information, both of which had correlations near 0.45 with Mechanical Comprehension. Overall, the dimensions that were not retained had correlations ranging from 0.45 to 0.93 with the dimensions that were retained.

Outcome measures included behaviorally anchored performance rating scales, which were completed by supervisors. The performance ratings were assessed on 3 dimensions. *Effort* was a 3-item measure (internal consistency reliability of 0.89) assessing Soldiers’ persistence and initiative demonstrated when completing study, practice, preparation, and participation activities during training. *Discipline* was a 5-item measure (internal consistency reliability of 0.90) assessing Soldiers’ willingness to follow directions and regulations, and to behave in a manner consistent with the Army’s Core Values (e.g., showing up on time and showing proper respect for superiors). *Peer Leadership* (internal consistency reliability of 0.87) was a 3-item measure assessing Soldier’s proficiency in leading their peers (e.g., gaining the cooperation of peers, taking on leader roles as assigned, and giving clear directions to peers) when assigned to a leadership position.

### 3.2. Analytic Approach

Data analyses were conducted using the Statistical Package for the Social Sciences (SPSS) and Mplus software program (Muthén and Muthén 2017). First, the data were analyzed using descriptive statistics that examined the means, standard deviations, and correlations across the relevant variables. Next, Latent Profile Analysis (LPA) was used to derive categorical latent variables, which represent classes of individuals who share similar profiles (Lanza et al. 2003). After assessing model assumptions (Bauer 2007), determination of the *optimal* number of classes/profiles in LPA was conducted. This process required the specification and testing of multiple class solutions (1-class, 2-class, 3-class, etc.). Each class solution was examined using two types of estimation procedures, as implemented by the Mplus software: (1) a full-information maximum likelihood approach and (2) a Bayesian approach. From these models, the designation of the “best-fitting” model was determined. The Lo–Mendell–Rubin Adjusted Likelihood Ratio Test (LMRT; Lo et al. 2001) was developed as an inferential statistical test to determine model fit. The LMRT provided an indication of statistically significant improvement in fit for a model with *k* latent classes/profiles—as compared to a model with *k-1* latent classes/profiles—by approximating the differences between two log likelihood values (instead of using the χ^2^ distribution). Thus, a significant LMRT test indicated that a more complex model (e.g., 3-class) provided superior fit to a less complex model (e.g., 2-class). A second inferential test that was used when evaluating class enumeration was the Bootstrapped Likelihood Ratio Test (BLRT; Arminger et al. 1999). Rather than approximating a log likelihood difference distribution like the LMRT does, the BLRT in effect estimates a “difference” distribution by which different models can be compared, through the use of repeated sampling methods.

A number of fit indicators based on information criteria were also employed, and these include the Akaike Information Criteria (AIC; Akaike 1974), the Bayesian Information Criterion (BIC; Schwarz 1978), and the sample size-adjusted BIC (sBIC; Sclove 1987). Each of these information criteria is based on the log likelihood function for individual models (rather than comparing two log likelihood values, as LMRT and BLRT do). In addition, another statistical indicator of class enumeration is entropy (Ramaswamy et al. 1993). Entropy is a measure of how well classes or profiles can be distinguished, or the percentage of individuals in the sample that were correctly classified given the specific class model. In contrast to other statistical grouping approaches, such as cluster analysis, individuals in LPA are assigned a *posterior probability* for each class/profile, rather than outright assignment to just one class/profile. Entropy is the aggregate of these posterior probabilities, with values greater than 80% being considered noteworthy.

The interpretability of each class/profile was used to facilitate the determination of whether or not a specific class solution is consistent with past theory and empirical research. Two primary model parameters are useful in this regard in LPA: (1) latent class probabilities (LCP), and (2) conditional response means (CRM). CRMs are analogous to factor loadings (Lanza et al. 2003), and they refer to the mean for each observed variable *within* a latent profile. These classes/profiles are then substantively characterized by interpreting responses both within and between classes. The CRMs are good indications of which observed variables within and between classes best identify the separate classes or profiles. Once classes/profiles are substantively interpreted, the probability or the proportion of cases within each class/profile helps identify the prevalence of class/profile membership.

## 4. Results

Descriptive statistics and correlations were calculated among the study variables (see Table 1). Latent profile analyses were conducted to identify the best-fitting profile solution. Table 2 shows the results for the profile solutions that ranged from two classes to eight classes. The results indicated that the Akaike Information Criteria (AIC), Bayesian Information Criterion (BIC), and the sample-adjusted BIC (sBIC) generally decreased as the number of profiles increased from two to eight. The largest decrease in the sBIC was between the 4-profile solution (sBIC = 62,228.96) and 5-profile solution (sBIC = 61,859.75), and the sBIC remained relatively stable from the 4-profile solution to 8-profile solution (sBIC = 61,699.32). In addition, although the 3-profile solution presented the largest entropy value (0.78), both the Lo–Mendell–Rubin Adjusted Likelihood Ratio Test (LMRT) and the Bootstrapped Likelihood Ratio Test (BLRT) were significant, suggesting the 3-profile solution was not a better fit of the data than the 2-profile solution. As such, additional profile solutions were examined. The 6-profile solution presented with a non-significant LMRT, suggesting that the 5-profile solution was the best fitting model for the data.

**Figure 1:**
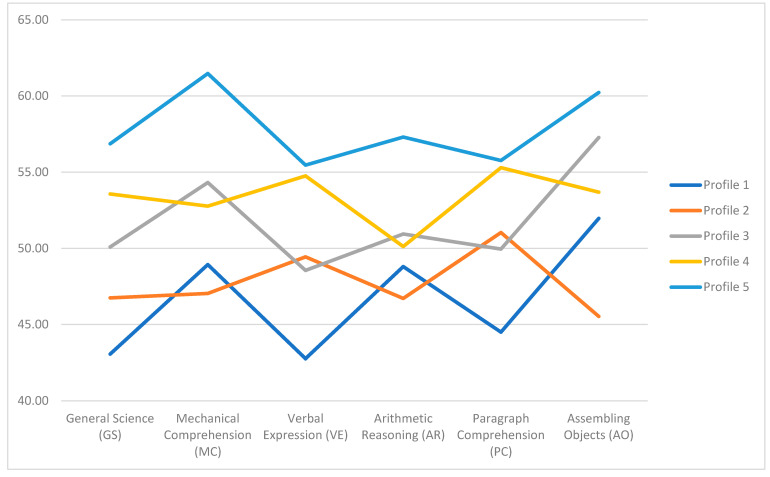
Cognitive Ability Classes by Average Armed Service Vocational Aptitude Battery Dimension. Note. Profile 1 = Arithmetic Reasoning/Assembling Objects; Profile 2 = Verbal Expression and Paragraph Comprehension; Profile 3 = Math Knowledge/Assembling Objects; Profile 4 = Verbal Expression, Paragraph Comprehension, and Assembling Objects; Profile 5 = Uniformly High including General Science, Math, and Verbal.

The 5-profile solution was further examined for theoretical and practical significance using conditional response means (CRMs). Based on fit statistics, meaningfulness of the CRMs, the minimum number of individuals per class, and distinct relationships between the profiles and performance outcomes, the 5-profile solution was determined to best represent the underlying data. Notably, profiles within the 5-profile solution had overlapping mean structures, suggesting the response patterns within each profile had unique interactional effects (see Figure 1). Additionally, the 5-profile solution showed evidence of acceptable classification probabilities (see Table 3), suggesting the profiles had reliable classification rates. In Table 3, the accuracy classification probabilities for each of the classes are in the diagonal (higher and closer to 1 is better). The average for the diagonal entries was 0.81.

Each profile highlighted relative cognitive ability strengths based on six dimensions of the ASVAB: General Science, Mechanical Comprehension, Verbal Expression, Arithmetic Reasoning, Paragraph Comprehension, and Assembling Objects. Table 4 shows the overall sample means and conditional response means for the 5-class solution. Profile 1 (13% of the sample) was labeled *Arithmetic Reasoning/Assembling Objects*. Profile 2 (15% of the sample) was labeled *Verbal Expression and Paragraph Comprehension*. Profile 3 (35% of the sample) was labeled *Math Knowledge/Assembling Objects*. Profile 4 (20% of the sample) was labeled *Verbal Expression, Paragraph Comprehension, Assembling Objects,* and Profile 5 (17% of the sample) was labeled *Uniformly High, including General Science, Math, and Verbal*.

Next, a 3-step mixture modeling approach (Asparouhov and Muthén 2015) was conducted to examine if there were significant differences between the cognitive profiles and performance ratings. As shown in Table 5, significant differences were found across classes for the Effort (*M* = 3.51; *SD* = 0.74), Discipline (*M* = 3.77; *SD* = 0.73), and the Peer Leadership (*M* = 3.39; *SD* = 0.79) ratings. Those classified as Profile 1 received significantly lower Effort ratings (*M* = 3.40; *SD* = 0.73) than Profile 3 (*p* < 0.01), Profile 4 (*p* < 0.05), and Profile 5 (*p* < 0.01). No significant differences in Effort ratings were found between those classified as Profile 3 (*M* = 3.55; *SD* = 0.71), Profile 4 (*M* = 3.56; *SD* = 0.69), and Profile 5 (*M* = 3.68; *SD* = 0.72). Regarding Discipline, those classified as Profile 2 (*M* = 3.53; *SD* = 0.80) reported significantly (*p* < 0.01) lower Discipline ratings than Profile 1 (*M* = 3.66; *SD* = 0.71, *p* < 0.01), Profile 3 (*M* = 3.79, *SD* = 0.72; *p* < 0.05), Profile 4 ((*M* = 3.83; *SD* = 0.69, *p* < 0.01), and Profile 5 (*M* = 3.94; *SD* = 0.69, *p* < 0.01). Those classified as Profile 4 (*M* = 3.83; *SD* = 0.69) and Profile 5 (*M* = 3.94; *SD* = 0.69) had significantly (*p* < 0.01) higher Discipline ratings as compared to those classified as Profile 1 (*M* = 3.66; *SD* = 0.71). Finally, for Peer Leadership, those classified as Profile 5 reported significantly (*p* < 0.05) higher ratings (*M* = 3.56; *SD* = 0.78) than those classified as Profile 1 (*M* = 3.31; *SD* = 0.73, *p* < 0.01), Profile 2 (*M* = 3.22; *SD* = 0.85, *p* < 0.01), and Profile 4 (*M* = 3.38; *SD* = 0.77, *p* < 0.05), but not Profile 3 (*M* = 3.42; *SD* = 0.78).

## 5. Discussion

This paper utilized a person-centered approach, multiple cognitive ability dimensions, and latent profile analysis to investigate multivariate relationships between cognitive ability dimensions. Specifically, this study proposed that cognitive ability can be considered more holistically across multiple dimensions, instead of relying on an overall “g” factor. This approach was investigated using a person-centered analysis, in which profiles were generated using latent profile analysis. The results identified five distinct profiles or classes, which differed significantly across the three types of supervisor ratings. Thus, in terms of the research questions proposed earlier in this paper, this study indicated that the Latent Profile Analyses provided evidence of multiple classes/profiles based on cognitive ability dimensions. Further, this study indicated that the performance outcomes differed across the cognitive ability profiles.

As noted earlier in this paper, research on cognitive ability from I–O psychologists has not made much progress in many years, and new perspectives on cognitive ability are needed. This paper provides one new way of examining cognitive ability in terms of using a person-centered approach that considers multiple dimensions of cognitive ability simultaneously; that is, applicants and workers are not just described and judged by their overall cognitive ability score, or even by separate specific cognitive ability dimensions. Instead, individuals can be viewed as having patterns of cognitive abilities that interact simultaneously (rather than using a variable-centered approach that considers independent cognitive dimensions). Kaufman (2019) noted that if one really wants to understand the complexities of a person’s intelligence, one can do much better than simply looking at a person’s overall IQ score. Kaufman argued that the overall IQ or intelligence score doesn’t offer nearly as much information as the pattern of meaningful cognitive dimensions. The current study is one of the first to empirically support this argument.

This research has extended the work on person-centered approaches to the cognitive domain. In this study, the LPA results described five profiles that have differing patterns across the cognitive dimensions. More specifically, each of the five profiles had different sets of cognitive strengths. Profile 5 comprised individuals who scored highly across all cognitive dimensions; however, not all applicants or employees can be above average. Thus, it is important to examine the other cognitive profiles or classes. Individuals in Profile 4 were relatively high on Verbal Expression, Paragraph Comprehension, and Assembling Objects. Although they did not have the very high cognitive ability across all dimensions as those in Profile 5, those in Profile 4 nevertheless had similar average scores on both Effort and Discipline performance ratings. As another example, those in Profile 3 (who scored highly in Math Knowledge and Assembling Objects, but not as high in Verbal Expression and Paragraph Comprehension) received high Peer Leadership ratings (which were not significantly different from the Uniformly High members of Profile 5).

It is notable that the profiles did not show a simple linear pattern when they are examined across the different performance ratings. Specifically, across the different performance ratings, the highest ability profile was not necessarily statistically significantly different from some of the other profiles. This finding is unique compared to how general cognitive abilities are typically discussed in the literature, which very often assumes that more cognitive ability is always better (e.g., Schmidt 2002). Thus, the results of this study provide another indication that the consideration of cognitive profiles using the person-centered approach can provide an alternative perspective to the prevailing view.

### Strengths, Limitations, and Future Research

A strength of the current study is that it is the first to examine relationships among multiple cognitive dimensions using a person-centered approach and LPA. One limitation with the current study is the heavily male sample. A second limitation is that this study only investigated cognitive ability dimensions as part of the latent profile analysis. Future research should include both cognitive ability and personality dimensions in a latent profile analysis to investigate patterns and profiles across these two important ways in which individuals differ. Following the research on personality profiles identified using the person-centered approach (Asendorpf 2015; Donnellan and Robins 2010), future research should examine whether the cognitive profiles identified in the current study can be replicated in other samples. To our knowledge, no other studies have been conducted using both cognitive ability dimensions and a person-centered approach; thus, the profiles identified will need to be examined with other samples and other cognitive measures. This study used six dimensions from the ASVAB (General Science, Mechanical Comprehension, Verbal Expression, Arithmetic Reasoning, Paragraph Comprehension, and Assembling Objects). Although these six dimensions include both ability and knowledge subtests, other cognitive ability measures differ somewhat on which dimensions they measure. For example, the Wechsler Adult Intelligence Scale—Fourth Edition (WAIS-IV) includes indexes on four factors: verbal comprehension, perceptual reasoning (spatial reasoning), working memory (including arithmetic and quantitative reasoning), and processing speed (Holdnack 2019). As another example, the Stanford–Binet intelligence test assesses five factors: knowledge, quantitative reasoning, visual–spatial processing, working memory, and fluid reasoning (Bain and Allin 2005). Roberts et al. (2000) examined the ASVAB’s factorial structure in relation to the theory of fluid and crystallized intelligence and Carroll’s (1993) three-stratum model that includes narrow abilities (Stratum 1), broad abilities (Stratum 2), and general cognitive abilities (Stratum 3). They found that the ASVAB measured primarily crystallized intelligence, and thus did not measure fluid intelligence or memory as much as other well-known cognitive ability tests. Adopting a person-centered approach and considering important theoretical models of cognitive ability (Carroll 1993; McGrew 2005) while examining additional samples and other cognitive measures will help researchers to develop further understanding of patterns across cognitive dimensions.

Wai et al. (2009) found that spatial ability plays a critical role in developing expertise in STEM (science, technology, engineering, and mathematics) jobs. They recommended that including spatial ability in modern talent searches would identify adolescents with potential for STEM jobs. The current study found that combinations of cognitive abilities (including spatial ability, which is assessed by the Assembling Objects dimension of the ASVAB) are related to work outcomes. Future research should further investigate how spatial ability works in combination with other cognitive dimensions to predict performance outcomes.

Oswald and Hough (2012) noted that research has documented that, even within range-restricted samples of gifted young students, higher SAT scores predict higher levels of career outcomes in middle age (Wai et al. 2005). They noted that researchers could investigate similar questions within occupational samples. For example, how does talent distinguish itself (are there different patterns of cognitive abilities?) within high-ability professionals, even within the same occupation? Oswald and Hough further suggested that knowing more about how highly talented individuals develop could have important implications for understanding and managing talent and varying cognitive abilities in the workforce. Future research should investigate these issues with profiles of multiple cognitive abilities along with incorporating antecedents of these cognitive profiles.

Overall, this study used multiple cognitive ability dimensions and LPA to identify five latent classes. The classes were found to have significant differences across a number of supervisor performance ratings. Future research should continue to use a person-centered approach to investigate cognitive dimensions as part of latent classes that may be predictive of performance outcomes.

## Figures and Tables

**Table 1:** Correlations among study variables (n = 1681).

Variable	*M*	*SD*	1	2	3	4	5	6
1. General Science	50.54	6.63						
2. Mechanical Comprehension	53.47	6.57	0.40 **					
3. Verbal Expression	50.35	4.81	0.59 **	0.34 **				
4. Arithmetic Reasoning	50.98	5.73	0.27 **	0.36 **	0.24 **			
5. Paragraph Comprehension	51.47	4.78	0.37 **	0.29 **	0.71 **	0.27 **		
6. Assembling Objects	54.65	7.29	0.21 **	0.42 **	0.16 **	0.34 **	0.18 **	
7. Gender	0.09	0.29	−0.04	−0.20 **	0.06 *	−0.05	0.07 **	−0.03

*Note.* * indicates *p* < 0.05. ** indicates *p* < 0.01. Gender is coded 0 = male, 1 = female.

**Table 2:** Model Fit Indices for the Latent Profile Analysis Solutions (n = 1681).

Solution	LMRT (*p*)	BLRT (*p*)	AIC	BIC	sBIC	Entropy	%’s for Classes	No. Parameters
2 class	169.52 (<0.001)	<0.001	62,706.91	62,810.03	62,749.67	0.75	57, 43	19
3 class	540.07 (<0.001)	<0.001	62,170.45	62,311.56	62,228.96	0.78	16, 52, 32	26
4 class	216.38 (0.024)	<0.001	61,963.91	62,143.01	62,038.17	0.71	15, 20, 33, 33	33
5 class	204.25 (<0.001)	<0.001	61,769.74	61,986.82	61,859.75	0.71	13, 15, 35, 20, 17	40
6 class	86.57 (0.296)	<0.001	61,695.51	61,950.58	61,801.27	0.70	6, 16, 31, 17, 14, 16	47
7 class	73.13 (0.206)	<0.001	61,634.97	61,928.04	61,756.49	0.72	3, 3, 18, 18, 14, 28, 16	54
8 class	88.01 (0.316)	<0.001	61,562.05	61,893.10	61,699.32	0.72	2, 11, 12, 7, 14, 30, 8, 14	61

*Note.* LMRT = Lo–Mendell–Rubin Test; BLRT = Bootstrap Likelihood Ratio Test; AIC = Akaike Information Criterion; BIC = Bayesian Information Criterion; sBIC = Sample Size-Adjusted Bayesian Information Criterion.

**Table 3:** Classification Probabilities for the Most Likely Latent Class Membership (Column) by Latent Class (Row).

	1	2	3	4	5
1	**0.86**	0.04	0.10	0.00	0.00
2	0.04	**0.75**	0.17	0.04	0.00
3	0.03	0.06	**0.84**	0.05	0.02
4	0.00	0.04	0.11	**0.76**	0.09
5	0.00	0.00	0.03	0.12	**0.85**

*Note.* 1 = Arithmetic Reasoning/Assembling Objects; 2 = Verbal Expression and Paragraph Comprehension; 3 = Math Knowledge/Assembling Objects; 4 = Verbal Expression, Paragraph Comprehension, Assembling Objects; 5 = Uniformly High including General Science, Math, and Verbal. The accuracy classification probabilities for each of the classes are in the diagonal.

**Table 4:** Overall Sample Means and Conditional Response Means (CRMs for 5-Class Solution (n = 1681)).

			Profile 1	Profile 2	Profile 3	Profile 4	Profile 5
Sample Size			n = 218	n = 250	n = 591	n = 330	n = 292
% of Sample			13%	15%	35%	20%	17%
	Overall Mean	S.D.	Conditional Response Means (CRM)				
General Science (GS)	50.54	6.63	43.06	46.74	50.09	53.57	56.86
Mechanical Comprehension (MC)	53.47	6.57	48.93	47.04	54.31	52.77	61.48
Verbal Expression (VE)	50.35	4.81	42.77	49.44	48.55	54.75	55.46
Arithmetic Reasoning (AR)	50.98	5.73	48.80	46.71	50.94	50.12	57.30
Paragraph Comprehension (PC)	51.47	4.78	44.50	51.04	49.95	55.30	55.76
Assembling Objects (AO)	54.65	7.29	51.97	45.54	57.27	53.69	60.23

*Note.* Profile 1 = Arithmetic Reasoning/Assembling Objects; Profile 2 = Verbal Expression/Paragraph Comprehension; Profile 3 = Math Knowledge/Assembling Objects; Profile 4 = Verbal Expression, Paragraph Comprehension, Assembling Objects; Profile 5 = Uniformly High including General Science, Math, and Verbal.

**Table 5:** Comparison of Classes on Performance Ratings (n = 1681).

Outcome	*Χ* ^2^			*M* (*SD*)		
		Profile 1	Profile 2	Profile 3	Profile 4	Profile 5
Effort Rating	56.56 **	3.40 (0.73) ^a^	3.26 (0.84) ^b^	3.55 (0.71) ^c^	3.56 (0.69 ^c^	3.68 (0.72) ^c^
Discipline Rating	65.92 **	3.66 (0.71) ^b^	3.53 (0.80) ^a^	3.79 (0.72) ^b,c^	3.83 (0.69) ^c^	3.94 (0.69) ^c^
Peer Leadership	26.45 **	3.31 (0.73) ^a^	3.22 (0.85) ^a,b^	3.42 (0.78) ^a,c^	3.38 (0.77) ^a,b,c^	3.56 (0.78) ^c^

*Note*. Means in the same row that do not share a superscript are significantly (*p* < 0.05) different from each other. Profile 1 = Arithmetic Reasoning/Assembling Objects; Profile 2 = Verbal Expression and Paragraph Comprehension; Profile 3 = Math Knowledge/Assembling Objects; Profile 4 = Verbal Expression, Paragraph Comprehension, Assembling Objects; Profile 5 = Uniformly High including General Science, Math, and Verbal. ** *p* < 0.01.

## Data Availability

Data used in this study are unavailable due to privacy restrictions.
